# Medicinal Plants

**DOI:** 10.3390/plants10071355

**Published:** 2021-07-02

**Authors:** Mariangela Marrelli

**Affiliations:** Department of Pharmacy, Health and Nutritional Sciences, University of Calabria, Rende, 87036 Cosenza, Italy; mariangela.marrelli@unical.it

Medicinal plants represent the most ancient form of medication, used for thousands of years in traditional medicine in many countries around the world. The empirical knowledge about their beneficial effects was transmitted over the centuries within human communities [1].

Natural products play a pivotal role as a source of drug compounds and, currently, a number of modern drugs which are derived from traditional herbal medicine are used in modern pharmacotherapy [2].

The extraction procedure is a crucial step in the study of the bioactive molecules from plant sources. Currently, in addition to more traditional techniques, modern extraction methods are being utilized, such as ultrasound-assisted and supercritical fluid extraction methods [3]. Moreover, the development of advanced tools for the qualitative and quantitative assessment of phytochemicals, such as high-performance liquid chromatography (HPLC) and liquid chromatography/mass spectrometry (LC/MS), significantly improved phytochemical investigation [4].

On the other hand, the biological properties of many plant species traditionally utilized together with their bioactive components have been elucidated until now. The more classical bioassay-guided natural drug discovery process and the modern processes, including high-throughput screening [4,5], and even the new reverse pharmacognosy approach [6], allowed the identification of a great number of bioactive phytochemicals.

Nevertheless, medicinal plants still have a hopeful future, as the phytochemical composition and the potential health benefits of many species have not yet been studied or still need to be more deeply investigated [4].

This Special Issue consists of 13 papers dealing with the phytochemical investigation and biological properties of medicinal plants. It includes original articles and reviews to offer to the readers updated information about the bioactive principles of some interesting plant species.

Gebashe and coworkers described the phytochemical profile of 13 medicinal grasses (Poaceae family) traditionally used in South Africa [7]. Among the investigated species, methanolic extracts from *Cymbopogon nardus* L. and *Cenchrus ciliaris* L. roots appeared particularly interesting for the abundance of the total soluble phenolics and flavonoids. The first species also showed the highest concentrations of condensed tannins and total iridoids. Some phenolic compounds, such as ferulic, salicylic, *p*-coumaric and vanillic acids were also quantified. Overall, the analyzed grass species showed a good antioxidant activity, as verified using the DPPH test and the ferric-reducing-power assay, thus suggesting that these species could be a source of interesting phytochemicals potentially useful for therapeutic purposes.

The ethnomedicinal study by Rahman and colleagues documented the traditional use of herbal teas for the treatment of various diseases in the Moonor Valley, Pakistan, whose local communities still strongly rely on their popular medicine [8]. Interviews of the local inhabitants allowed to identify 27 plant species, mainly herbs, but also shrubs and trees, which were used for the treatment of different diseases, with diarrhea and gas troubles being the most frequent disorders. Interestingly, the authors found that more than half of these medicinal uses were new to the scientific literature.

Ethnobotanical studies mainly focus on plant species traditionally used for therapeutic applications or as food, while ornamental plants are usually taken into account to a minor extent. However, the wide use of a plant for decorative purposes may also constitute the starting point for the study of further potential uses [9]. This is the case of *Abeliophyllum distichum* Nakai, commonly known as white forsythia. This ornamental plant that is endemic to Korea was investigated by Yoo and coworkers for its anticancer potential [10]. The methanolic extract of the leaves showed a concentration-dependent cytotoxicity against the human melanoma SK-MEL-2 cell line. Necrosis and apoptosis were both detected through the Annexin V/propidium iodide (PI) staining assay and the analysis of caspase activity. Furthermore, HPLC analyses allowed to identify 18 polyphenolic compounds, with taxifolin, rutin and luteolin being the most abundant components.

The paper by Sharifi-Rad and colleagues reported the antifungal and antibacterial properties of different extracts of *Nepeta juncea* Benth. [11]. The methanolic extract of the leaves showed the best activity, with an MIC value of 25 µg/mL for *Candida albicans*. The methanolic extracts from the leaves, flowers and roots were active against *Staphylococcus aureus* and *Bacillus cereus*, with MIC values equal to 25, 25 and 50 µg/mL, respectively. A screening of some secondary metabolite main classes, such as phenols, flavonoids, anthocyanins and tannins, was also carried out, and a number of phytochemicals were identified using gas chromatography-mass spectrometry (GC-MS), with 1,8-cineole, β-pinene, terpinen-4-ol, α-terpineol and 4aα-7α-7aα-nepetalactone being the major compounds.

Ilina and coworkers described the phytochemical profile of *Galium eparine* L. (Rubiaceae) [12]. Hydroalcoholic extracts were investigated with UHPLC-DAD-MS/MS analyses. Iridoids and polyphenols were the major detected phytochemicals, and the flavonoid derivative quercetin 3-*O*-rhamnoglucoside-7-*O*-glucoside was identified for the first time in a *Galium* species. The authors also verified the immunomodulatory activity of the extracts using the lymphocyte blast transformation in vitro model. All *G. aparine* samples stimulated the transformational activity of peripheral blood mononuclear cells, with the 96% ethanolic extract being the most active.

The *Allium* genus (Amaryllidaceae) comprises a number of species that have been used since ancient times as food and spices, but that have also been utilized in many cultures for medicinal purposes [13]. These species mainly contain organosulfur compounds, polyphenols and saponins [14]. *A. cepa* is widely cultivated almost all over the world and contains a number of interesting phytonutrients not only from a nutritional point of view, but also for their potential therapeutic applications [15]. Fredotović and colleagues investigated the antiproliferative activity of two *Allium* species, the triploid hybrid onion, *Allium cornutum* Clementi ex Visiani, 1842, and common onion, *Allium cepa* L. [16]. The hydroalcoholic extracts were tested on three different human cancer cell lines. The best results were obtained against HeLa and HCT116 cells. DNA fragmentation assay, DAPI staining and real time PCR analysis were also carried out, and obtained results confirmed that both onion extracts induced apoptosis in HeLa cells.

The paper by Rehman and Rather describes the ability of the natural flavonoid myricetin to counteract cisplatin-induced toxicity [17]. Cisplatin is one of the most potent anticancer drugs, but its use causes a number of side effects, such as nephrotoxicity, neurotoxicity, hepatotoxicity and gastrointestinal side effects, mainly due to a strong production of reactive oxygen species (ROS) and the consequent oxidative stress [18]. The protective effects of myricetin in cisplatin-induced toxicity on the colon were assessed in vivo on Wistar male rats treated orally with this flavonoid for 14 days at two doses (25 and 50 mg/kg of body weight) [17]. The obtained results demonstrated that myricetin was able to improve the anti-oxidant status, by ameliorating cisplatin-induced lipid peroxidation, to protect tissues from induced damage, by controlling goblet cell disintegration, and to up-regulate inflammatory markers such as TNF-α, IL-6 and NF-kB.

Different plant extracts demonstrated a significant anti-arthritic [19,20] and anti-inflammatory potential [21,22]. Amodeo and colleagues reported the in vitro anti-inflammatory and antioxidant potential of alcoholic extracts and fractions of *Chenopodium album* L. and *Sisymbrium officinale* (L.) Scop., two edible plants with a long history of use in the folk medicine for the treatment of inflammatory disorders [23]. Both species demonstrated to inhibit the production of the pro-inflammatory mediator nitric oxide, which was verified in LPS-stimulated murine macrophages. The dichloromethane fraction of *C. album* was the most active sample. Furthermore, some samples showed anti-arthritic potential, inducing a significant protein anti-denaturation effect, which was verified on heat-treated bovine serum albumin.

An increasing number of studies have recently dealt with the potential role of edible and medicinal plants and different classes of phytochemicals in obesity prevention and management [24,25,26]. Obesity is one of the greatest public health concerns, and different comorbidities related to overweight have been identified, mainly diabetes, resulting in the common metabolic disorder known as metabolic syndrome [27]. The potential role of plant extracts or phytochemicals in diabetes management has been deeply reviewed as well [28,29].

The in vitro anti-obesity potential of wild *Lobularia maritima* (L.) Desv., a perennial herb endemic to Italy and traditionally used as food source, has been described in this special issue [30]. The alcoholic extract from the aerial parts and its fractions were tested for their ability to inhibit pancreatic lipase, a key enzyme responsible for the gastrointestinal digestion of dietary fats. An interesting biological potential was observed for the ethyl acetate fraction, suggesting that this plant could be a potential source of useful compounds for the treatment of obesity. The dichloromethane fraction also showed a good in vitro inhibitory activity towards the production of the pro-inflammatory mediator nitric oxide, which was verified on lipopolysaccharide (LPS)-stimulated RAW264.7 macrophages. Moreover, the phytochemical profile was investigated with GC-MS, HPLC-DAD, HPLC-HRMS and ESI-MS/MS analyses.

Faraone and coworkers reported the antidiabetic activity of *Azorella glabra* Wedd. (Apiaceae), an endemic Bolivian species [31]. The aerial parts ethanolic extract induced a concentration-dependent inhibition of amylase enzyme. Interestingly, almost all fractions induced a dose-dependent inhibition of α-glucosidase enzyme, with the crude extracts and its *n*-hexane and ethyl acetate fractions being even more effective than the positive control acarbose. Furthermore, the author assessed the ability of samples to inhibit acetylcholinesterase and butyrylcholinesterase enzymes, two important targets for the treatment of Parkinson’s and Alzheimer’s diseases. The phytochemical profile was also verified with LC-ESI-MS/MS analyses.

This Special Issue also contains three review articles. Natabanzi et al. reviewed the ethnobotanical value, phytochemistry and pharmacological properties of *Kigelia africana* (Lam.) Benth. (Bignoniaceae), a tree species endemic to Africa [32]. The fruits of this plant, as well as the stem bark, roots and leaves, have been widely used in traditional medicine preparations. Several phytochemical constituents have been identified, such as phenolic compounds, coumarins, terpenes, sterols, unsaturated fatty acids and, particularly, iridoids and quinones, which have been recognized in all plant parts. Antibacterial, antifungal, anti-inflammatory and antidiabetic activities are just some of the pharmacological properties highlighted for this plant species.

A review by Guerrero-Solano and colleagues provided an overview of the scientific reports about the antinociceptive effect of pomegranate (*Punica granatum* L.), utilized in traditional medicine for the treatment of different kinds of pain [33]. Recent preclinical and clinicals studies performed on different pain models support the use in popular medicine and seems to suggest that this species could have a potential application in the treatment of inflammatory, neuropathic and nociceptive pain. The antinociceptive effect of pomegranate was mainly related to contained polyphenols (ellagitannins, tannins, gallotannins, and free ellagic and gallic acids), flavonoids and fatty acids, but alkaloids were also taken into account.

Finally, Boukhatem and Setzer provided a review focusing on the potential therapeutic use of medicinal and aromatic plant extracts against coronaviruses infections [34]. The current global COVID-19 pandemic, caused by the novel severe acute respiratory syndrome coronavirus 2 (SARS-CoV-2), is one of the most discussed scientific topics and a huge public health challenge [35,36]. This review paper illustrated several antiviral chemical constituents extracted from herbal medicines and described their in vitro and in vivo effects against coronaviruses. The alkaloid lycorine, present in different Amaryllidaceae species, and glycyrrhizin from *Glycyrrhiza glabra* L. demonstrated, for example, a potent antiviral activity against SARS-CoV strains.

Overall, this Special Issue could attract the reader’s interest, as it contributes to the phytochemical investigation of interesting plant species and to highlighting new biological activities of plants extracts and natural compounds.
